# ARL-17477 is a dual inhibitor of NOS1 and the autophagic-lysosomal system that prevents tumor growth in vitro and in vivo

**DOI:** 10.1038/s41598-023-37797-4

**Published:** 2023-07-04

**Authors:** Teiko Komori Nomura, Satoshi Endo, Takuma Kuwano, Kazuya Fukasawa, Shigeo Takashima, Tomoki Todo, Kyoji Furuta, Takuhei Yamamoto, Eiichi Hinoi, Hiroko Koyama, Ryo Honda

**Affiliations:** 1 United Graduate School of Drug Discovery and Medical Information Sciences, Gifu University, Gifu, Japan; 2 Laboratory of Biochemistry, Department of Biopharmaceutical Sciences, Gifu Pharmaceutical University, Gifu, Japan; 3 Center for One Medicine Innovative Translational Research (COMIT), Gifu University, Gifu, Japan; 4 Laboratory of Pharmaceutical Analytical Chemistry, Gifu Pharmaceutical University, Gifu, Japan; 5 Laboratory of Pharmacology, Department of Bioactive Molecules, Gifu Pharmaceutical University, Gifu, Japan; 6 Division of Genomics Research, Life Science Research Center, Gifu University, Gifu, Japan; 7 Institute for Glyco-core Research (iGCORE), Gifu University, Gifu, Japan; 8 Division of Innovative Cancer Therapy, Advanced Clinical Research Center, The Institute of Medical Science, The University of Tokyo, Tokyo, Japan; 9 Department of Chemistry and Biomolecular Science, Faculty of Engineering, Gifu University, Gifu, Japan

**Keywords:** Cancer therapy, Autophagy, Pharmacology

## Abstract

ARL-17477 is a selective neuronal nitric oxide synthase (NOS1) inhibitor that has been used in many preclinical studies since its initial discovery in the 1990s. In the present study, we demonstrate that ARL-17477 exhibits a NOS1-independent pharmacological activity that involves inhibition of the autophagy-lysosomal system and prevents cancer growth in vitro and in vivo. Initially, we screened a chemical compound library for potential anticancer agents, and identified ARL-17477 with micromolar anticancer activity against a wide spectrum of cancers, preferentially affecting cancer stem-like cells and KRAS-mutant cancer cells. Interestingly, ARL-17477 also affected NOS1-knockout cells, suggesting the existence of a NOS1-independent anticancer mechanism. Analysis of cell signals and death markers revealed that LC3B-II, p62, and GABARAP-II protein levels were significantly increased by ARL-17477. Furthermore, ARL-17477 had a chemical structure similar to that of chloroquine, suggesting the inhibition of autophagic flux at the level of lysosomal fusion as an underlying anticancer mechanism. Consistently, ARL-17477 induced lysosomal membrane permeabilization, impaired protein aggregate clearance, and activated transcription factor EB and lysosomal biogenesis. Furthermore, in vivo ARL-17477 inhibited the tumor growth of KRAS-mutant cancer. Thus, ARL-17477 is a dual inhibitor of NOS1 and the autophagy-lysosomal system that could potentially be used as a cancer therapeutic.

## Introduction

ARL-17477, a synonym of AR-R 17,477, is a selective inhibitor of neuronal nitric oxide synthase (NOS1) that catalyzes the conversion of L-arginine to NO in neural tissue^1^. Since its initial discovery in the 1990s, this compound has been widely used as a treatment for stroke and traumatic brain injury in preclinical animal models^2–4^. Also, ARL-17477 has been used as a standard NOS1 inhibitor in vitro and in vivo^5–8^. However, the only known activity of ARL-17477 is related to NOS1 inhibition^9^; indeed, studies have yet to be conducted to investigate whether ARL-17477 exhibits a NOS1-independent pharmacological activity.

Macroautophagy (hereafter referred to as autophagy) is an essential cellular process that recycles dysfunctional organelles and protein aggregates for cell survival^10^. Autophagy begins with the formation of double-membrane vesicles, known as autophagosomes, which engulf dysfunctional organelles and protein aggregates. Subsequently, autophagosomes fuse with lysosomes to degrade the engulfed components. Lysosomes are acidic organelles (pH 4.5–5.0) that contain ~ 60 different degradative enzymes^11^. In addition to autophagy, lysosomes also play pivotal roles in many cellular processes, including lysosome-to-nucleus signaling, apoptosis, and endocytosis for the transport of materials across the cell membrane. The autophagy-lysosomal system regulates a variety of cellular functions; consequently, it has been explored as a therapeutic target in cancer treatment. Chloroquine (CQ) and its derivative hydroxychloroquine are the best known inhibitors of the autophagy-lysosomal system and showed therapeutic potency in several clinical trials^12–14^. Furthermore, the next generation of quinacrine derivatives are in development (Lys05 and DQ661), either as single agents or in combination therapies with conventional chemotherapeutics^15,16^.

In this study, we demonstrated that ARL-17477 inhibits the autophagic-lysosomal system to prevent tumor growth in vitro and in vivo. Initially, we screened a chemical compound library and identified ARL-17477 with micromolar anticancer activity against a wide spectrum of cancers. Next, we demonstrated that ARL-17477 inhibits the autophagic-lysosomal system in a manner analogous to CQ. Finally, systemic administration of ARL-17477 via an intravenous route was shown to significantly inhibit tumor growth in a xenograft mouse model. These findings demonstrate that ARL-17477 and lysosome-targeting therapy could be used as cancer therapeutics.

## Results

### Identification of ARL-17477 as an anticancer agent

**Figure 1:**
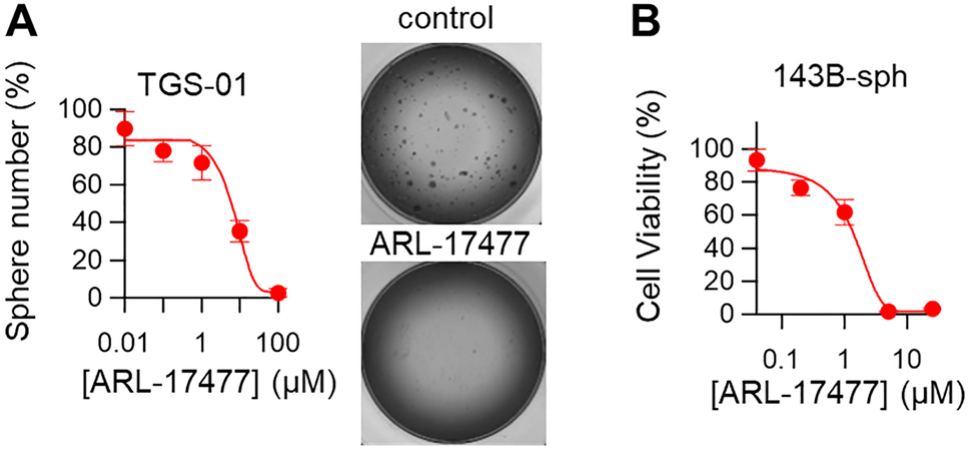
ARL-17477 affects cancer stem-like cells (CSCs). (**A**) Tumorsphere number of CSCs derived from glioma TGS-01 after 7 days of ARL-17477 treatment. IC_50_ of 4.4 ± 0.6 µM. Inset: representative images of the tumor sphere after 100-µM ARL-17477 treatment. (**B**) Cell viability curve for osteosarcoma 143B CSCs after 2 days of ARL-17477 treatment. IC_50_ of 1.1 ± 0.4 µM.

Initially, we constructed a library of chemical compounds that we synthesized in previous research projects, including the development of a positron emission tomography probe for NOS1. We screened this library for potential anticancer agents and identified ARL-17477 as a promising anticancer agent. ARL-17477 killed a wide spectrum of cancer cells, including colon cancer, lung cancer, pancreatic cancer, neuroblastoma and osteosarcoma, with IC_50_ values of 4.3 − 15.0 µM (Table 1). Interestingly, KRAS-mutant cancers were more vulnerable to ARL-17477 than KRAS-wild-type cancers. Moreover, ARL-17477 killed cancer stem-like cells (CSCs), derived from glioblastoma (TGS-01; Fig. 1A) and from osteosarcoma (143B-sph; Fig. 1B), with lower IC_50_ values of 4.4 and 1.1 µM, respectively. Thus, ARL-17477 is an anticancer agent targeting a wide variety of cancers, but it preferentially affects CSCs and KRAS-mutant cancers. Such cancer selectivity led us to explore the anticancer mechanism because CSCs and KRAS-mutant cancers are refractory to conventional chemotherapy and radiotherapy^17^.

**Table 1:** IC_50_ values of cell viability after 2 days of ARL-17477 treatment.

Cell lines	Cancer type	RAS mutations	IC_50_ (μM)
SW48	Colon cancer	None	15.0 ± 7.0
U251-MG	Neuroblastoma	None	13.0 ± 2.5
HCT116	Colon cancer	KRAS G13D	7.4 ± 1.3
A549	Lung cancer	KRAS G12S	6.8 ± 1.3
143B	Osteosarcoma	KRAS G12S	4.4 ± 1.0
MIA PaCa-2	Pancreatic cancer	KRAS G12C	4.3 ± 1.3

### Anticancer activity is NOS1-independent

Two previous studies already reported that ARL-17477 and its derivatives kill melanoma and chronic lymphocytic leukemia cells at low micromolar concentrations^18,19^. Neither studies investigated the mechanism of action, and the authors described that the anticancer activity was derived from NOS1 inhibition. However, NOS1 is mainly expressed in neurons, whereas ARL-17477 affected many non-neuronal cancer cells (Table 1). Moreover, whether NOS1 itself is essential for cell survival remains controversial^20–23^. Indeed, in the DepMap Portal database, no cancer cell line is considered dependent on NOS1 for survival^24^.

**Figure 2:**
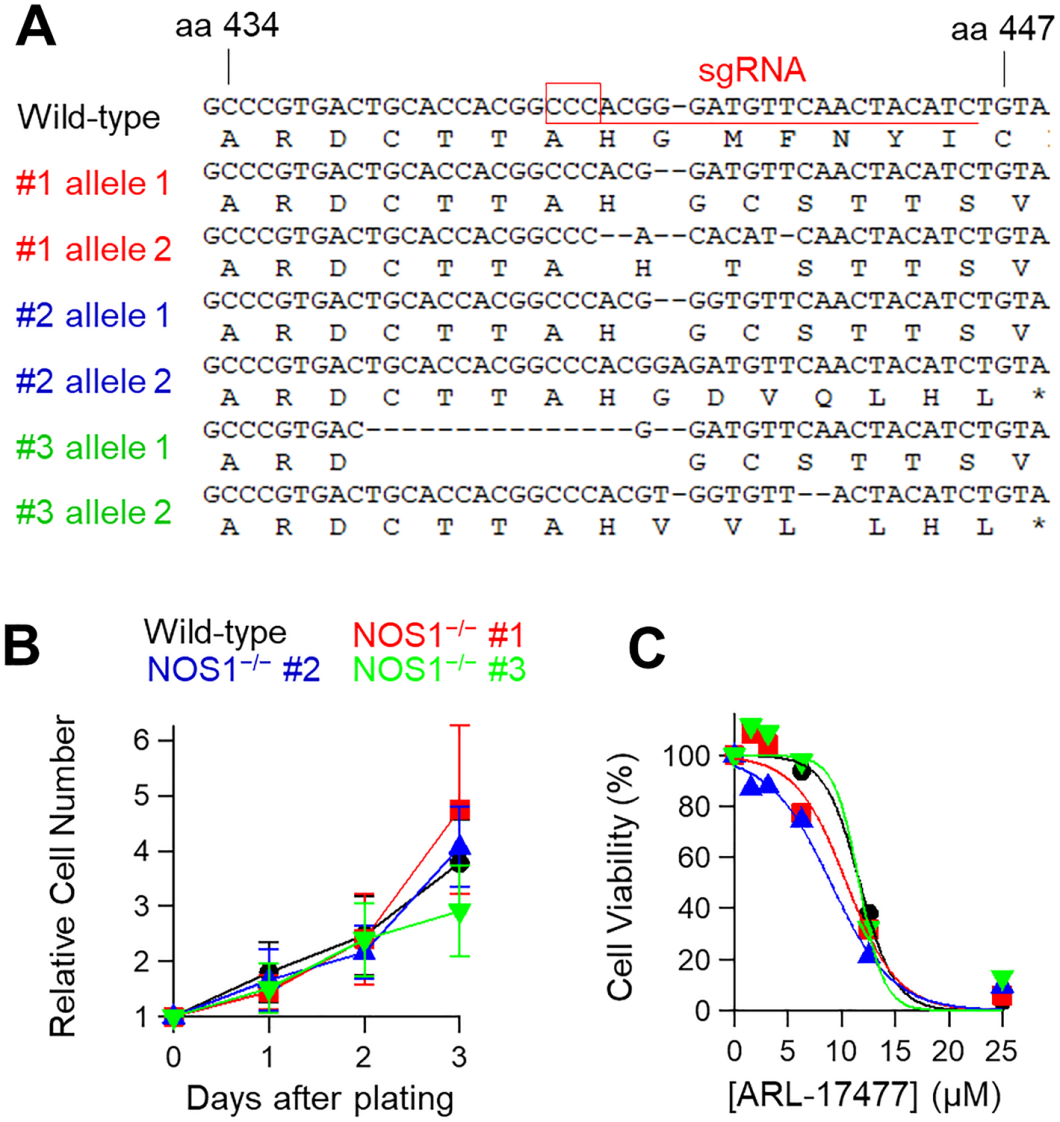
Anticancer action of ARL-17477 is NOS1-independent. (**A**) Alignment of *NOS1* gene sequences of wild-type and NOS1^−/−^ cell lines derived from U251-MG. (**B**) Growth curves of wild-type (black) and three NOS1^−/−^ cell lines (red, blue, and green). NOS1^−/−^ cell lines retained the normal growth rates with a doubling time of approximately 1.5 days. (**C**) Cell viability curves following 3 days of ARL-17477 treatment.

To examine whether NOS1 expression is required for the action of ARL-17477, we generated NOS1-knockout cell lines from U251-MG neuroblastoma using the CRISPR/Cas9 system. As a gene, *NOS1* comprises of 30 exons encoding a 1434-amino acid protein^25^. We designed an sgRNA targeting exon 7, which encodes the N-terminal oxygenase domain of NOS1. We generated three NOS1^−/−^ cell lines harboring frameshift mutations in the N-terminal part (Fig. 2A); however, the NOS1^−/−^ cell lines retained a normal growth rate similar to that of wild-type cell lines (Fig. 2B). Furthermore, immunoblot analysis did not detect the expression of NOS1 protein both in the wild-type and NOS1^−/−^ cell lysates (Fig. S1A), suggesting that the cancer cells express very low to no level of NOS1 protein. Moreover, ARL-17477 affected the NOS1^−/−^ cell lines at low micromolar concentrations (Fig. 2C). Thus, the anticancer activity of ARL-17477 is NOS1-independent.

### Analysis of cell survival signals and death markers

**Figure 3:**
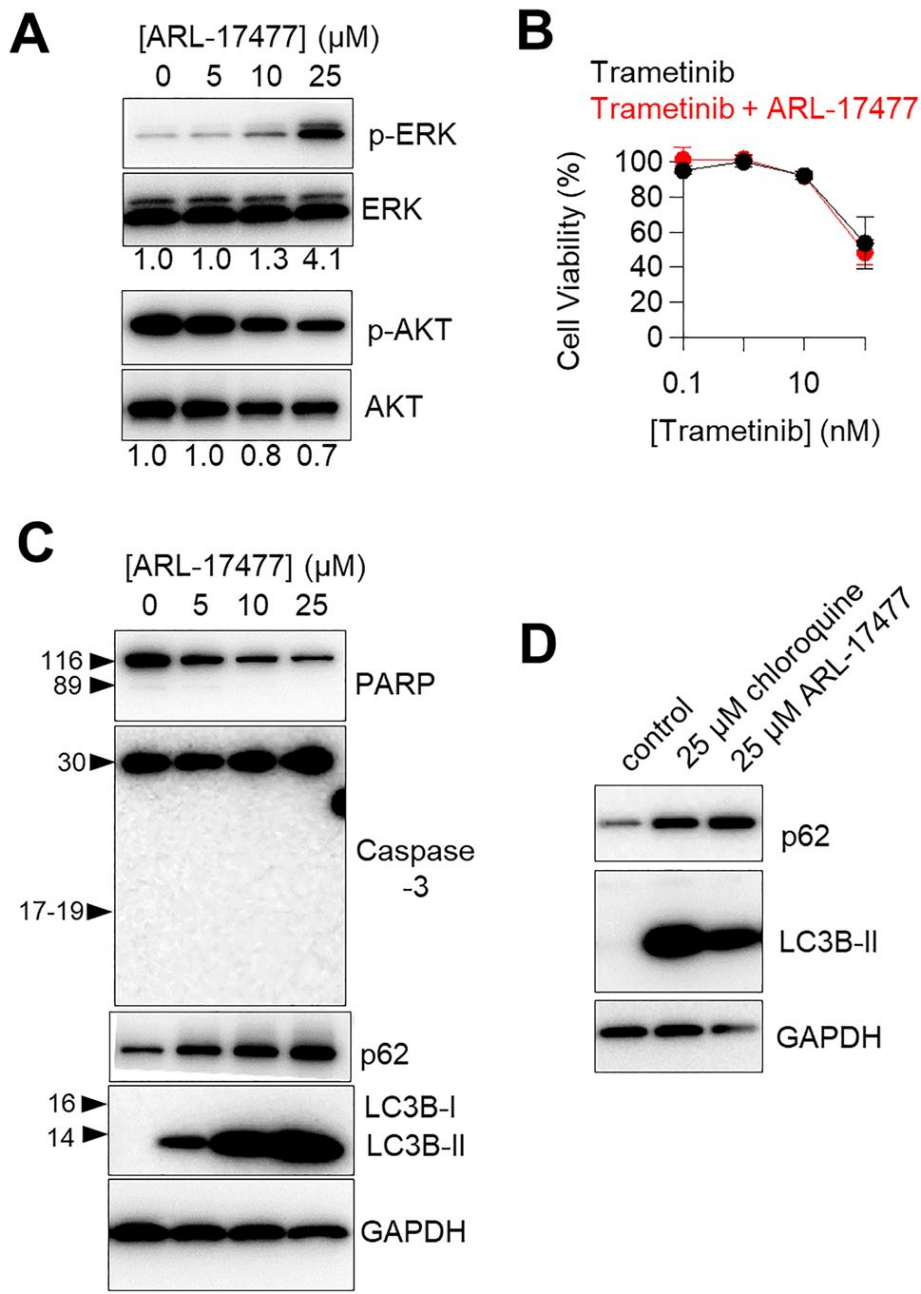
ARL-17477 increases LC3B-II and p62 protein expression levels. (**A**,**C**,**D**) Immunoblots of neuroblastoma U251-MG following 1 day of ARL-17477 or chloroquine treatment. The numbers below the figure A indicate the band intensities of phosphorylated protein levels relative to their unphosphorylated counterparts (the vehicle control was set to 1.0). Original blots are presented in Supplementary Fig. S3, which were cropped from different gels. (**B**) Cell viability curves of U251-MG after 3 days of treatment with trametinib only (black) or trametinib combined with 4-µM ARL-17477 (red; corresponding to IC_10_ concentration).

To explore the mechanism of NOS1-independent action, we examined whether ARL-17477 affects ERK and AKT pathways, which are involved in cell proliferation and survival. ARL-17477 induced hyperphosphorylation of ERK with a slight suppression of AKT phosphorylation (Fig. 3A). However, the ERK phosphorylation did not directly contribute to the anticancer mechanism, because a MEK inhibitor (trametinib) did not demonstrate a synergistic effect with ARL-17477 (Fig. 3B).

Next, we examined a series of cell death makers related to apoptosis and autophagy. ARL-17477 did not induce cleavage of caspase-3 or PARP (Fig. 3C), excluding the possibility of apoptosis. We observed reduction of full-length PARP, which might be a result of autophagic degradation and/or nonapoptotic cleavage of PARP^26^. By contrast, ARL-17477 significantly increased expression of the autophagy marker proteins LC3B-II and p62. This increase was observed in two tested cell lines after 6 h of treatment, and persisted for up to 6 days (Fig. S2). Of note, ARL-17477 also increased LC3B-II protein in the NOS1-knock cell lines (Fig. S1B), suggesting a NOS1-independent mechanism. Although assessment of LC3-II is not straightforward in the context of autophagy flux^27^, a concomitant increase in LC3-II and p62 protein expression was similar to that observed in CQ-treated cells (Fig. 3D). Thus, ARL-17477 might demonstrate a CQ-like activity, i.e. it might inhibit autophagic flux through inhibition of autophagosome-lysosome fusion^12^.

### Acidic environment of the lysosome is required for the action of ARL-17477

**Figure 4:**
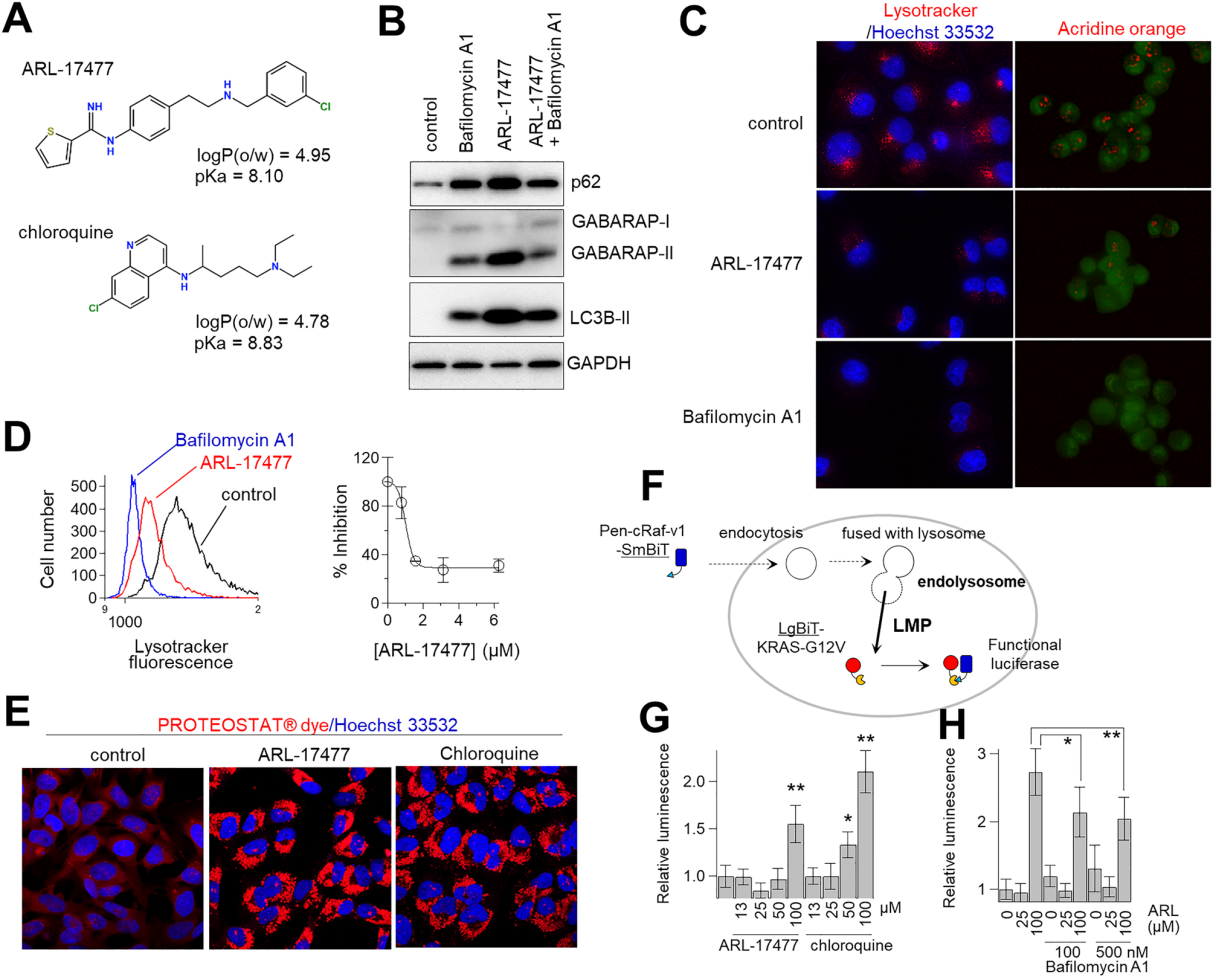
ARL-17477 induces lysosomal dysfunction and lysosomal membrane permeabilization. (**A**) Chemical structures of ARL-17477 and chloroquine with partition coefficients (logP) and acid dissociation constants (pKa). (**B**) Immunoblots of neuroblastoma U251-MG treated with 8-nM Bafilomycin A1 for 3 h, followed by 1 day of cotreatment with 25-µM ARL17477 and 8-nM Bafilomycin A1. Original blots are presented in Supplementary Fig. S4, which were cropped from different gels. (**C**) Fluorescence micrographs of MIA PaCa-2 cancer cells treated with 100-nM Bafilomycin A1 or 100-µM ARL-17477 for 30 min, followed by staining with acridine orange or Lysotracker Red IND-99. Red channels show stained lysosomes. (**D**) Left panel: histograms of MIA PaCa-2 cells stained with Lysotracker Red IND-99 after treatment with vehicle (black), 100-µM ARL17477 (red), and 100-nM Bafilomycin A1 (blue). Right panel: inhibition of Lysotracker staining as a function of ARL-17477 concentration; 100% inhibition was achieved using 100-nM Bafilomycin A1. (**E**) Fluorescence micrographs of MIA PaCa-2 treated with 2-µM ARL-17477 or 4-µM chloroquine for 5 days followed by staining with PROTEOSTAT dye. (**F**) Schematic view of a NanoBiT split luciferase assay for quantitative assessment of lysosomal membrane permeabilization. (**G**) Luciferase activity of LgBiT-KRAS-expressing cells treated with ARL-17477 or chloroquine for 1 h, followed by treatment with Pen-cRaf-v1-SmBiT for 1 h. (**H**) Luciferase activity of LgBiT-KRAS-expressing cells pretreated with Bafilomycin A1 for 30 min, followed by treatment with ARL-17477 for 30 min and Pen-cRaf-v1-SmBiT for 60 min. Statistical comparisons were performed using unpaired Student’s *t* tests for two tailed p value. *p < 0.05, **p < 0.01.

Figure 4A shows a comparison of the chemical structures of ARL-17477 and CQ. We found a common hydrophobic amine structure, similar hydrophobicity (logP), and similar acid dissociation constant (pKa) in these molecules. Previously, Nadanaciva et al.^28^ proposed that drugs with logP > 2 and a basic pKa of 6.5–11 are accumulated within the acidic environment of the lysosome and inhibit lysosomal function. To test whether the lysosomal acidity is essential for ARL-17477, we cotreated cells with ARL-17477 and Bafilomycin A1, which is known to increase lysosomal pH by inhibiting vacuolar-type H^+^-ATPase and thus was expected to inhibit the accumulation of ARL-17477 in lysosomes. As expected, Bafilomycin A1 alleviated the increase in LC3-II, p62, and GABARAP-II protein expression induced by ARL-17477 (Fig. 4B). Thus, the acidic environment of the lysosome is required for the accumulation and activity of ARL-17477.

### ARL-17477 inhibited lysosomal function

To examine whether ARL-17477 inhibits lysosomal function, we stained lysosomes with acridine orange and Lysotracker Red DND-99. Lysosomal staining has been widely used to measure lysosomal function, which is inhibited either by the loss of the acidic environment or by the induction of lysosomal membrane permeabilization (LMP)^29^. ARL-17477 markedly inhibited both types of staining (Fig. 4C). Quantitative analysis using fluorescent cytometry demonstrated that ARL-17477 inhibited lysosome staining even at 1 µM (Fig. 4D). Thus, ARL-17477 inhibits lysosomal function at a few micromolar concentrations.

The autophagy-lysosomal system is known to be essential for clearance of protein aggregates. To examine whether ARL-17477 impairs protein homeostasis, we stained aggresomes after prolonged treatment with ARL-17477. As a result, ARL-17477 significantly increased the number of aggresomes after 6 days of treatment (Fig. 4E). Thus, ARL-17477 inhibits the clearance of protein aggregates through inhibition of the autophagy-lysosomal system.

LMP is a hallmark of lysosomal dysfunction and contributes to lysosome-dependent cell death^30^. To assess the occurrence of LMP, we employed a luciferase assay that we developed previously^31^. In general, LMP is assessed using fluorescence microscopy by measuring the extent of cytosolic release of exogenous macromolecules (for example, fluorescent dextran: 1–70 kDa) from endolysosomes^32^. However, this method is qualitative; thus, in a previous study, we developed a luciferase assay that can quantitatively measure the extent of endolysosomal escape of macromolecules^31^ (Fig. 4F). This system relies on a NanoBiT split luciferase system and employs a SmBiT-tagged cell-permeable protein (Pen-cRaf-v1: 15 kDa)^33^. Because this protein is internalized by cells via endocytosis followed by endolysosomal escape, its internalization is enhanced by LMP. Accordingly, LMP enhances the cytosolic delivery of the protein and thereby increases the luciferase activity of the cells. In the present study, 100-µM ARL-17477 was shown to increase the luciferase activity by 1.5-fold (Fig. 4G). This activity was similar to that of CQ, which increased luciferase activity by 1.5–2.0 fold at the concentrations 50–100 µM. Furthermore, pretreatment with Bafilomycin A1 alleviated the increase in the luciferase activity induced by ARL-17477 (Fig. 4H). Thus, ARL-17477 induces LMP and allows extracellular macromolecules to translocate into the cytosol, similar to CQ. The observed discrepancy of the effective concentration between the NanoBiT assay (100 µM) and cell viability assay (1 − 15 µM) indicated that small leakage of lysosomal proteins into the cytosol triggers cell death^34^. Consistently, CQ induced LMP at the concentrations 50–100 µM (Fig. 4G), but its IC_50_ value for cell viability has been reported to be 10 − 20 µM^35^.

### Transcriptome microarray and gene set enrichment analysis

**Figure 5:**
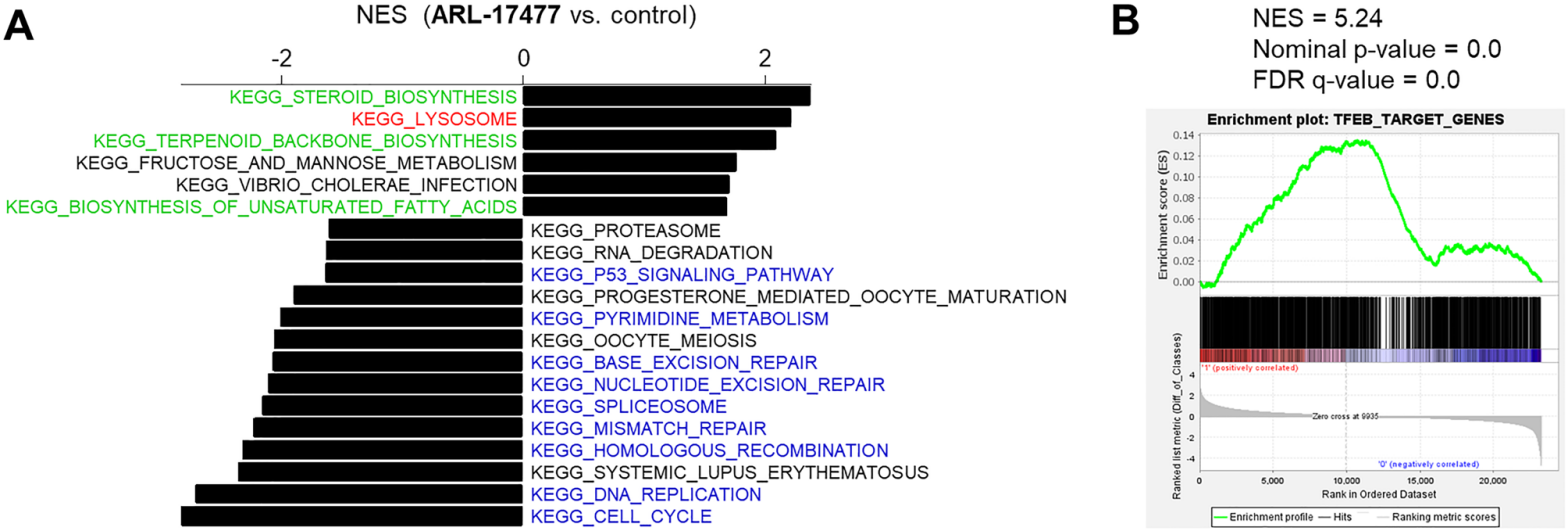
Transcriptome microarray analysis. (**A**) KEGG pathway gene sets enriched in ARL-17477-treated cells (positive values of NES) and untreated cells (negative values of NES). FDR values of < 0.25 with p < 0.01 were regarded as statistically significant. (**B**) Enrichment of TFEB-target genes in 25-µM ARL-17477-treated cells. The other enriched gene sets are listed in Table S1.

To comprehensively assess the pharmacological activity of ARL-17477, we employed transcriptome microarray and gene set enrichment analysis (GSEA). Initially, we queried the KEGG database to determine which biological pathways are altered by ARL-17477. As a result, gene sets related to lysosomes, cholesterol, and fatty acid synthesis were upregulated by ARL-17477 (Fig. 5A). This result was consistent with previous evidence showing that lysosome dysfunction induces a positive feedback regulation of lysosomal biogenesis through activation of transcription factor EB (TFEB)^36,37^. Because TFEB is a master regulator of autophagy-lysosome function controlling lysosomal biogenesis, we queried the gene sets comprised of 1128 transcription factor targets in MSigDB v7.5.1. As a result, the TFEB target gene set was ranked in the top five gene sets upregulated by ARL-17477 (Fig. 5B and Table S1). Meanwhile, gene sets downregulated by ARL-17477 were related to the cell cycle, pyrimidine biosynthesis, and DNA replication/repair, suggesting inhibition of cell proliferation (Fig. 5A). Thus, ARL-17477 induces (1) feedback activation of lysosomal biogenesis and (2) inhibition of cell proliferation.

### ARL-17477 inhibited tumor growth in vivo

**Figure 6:**
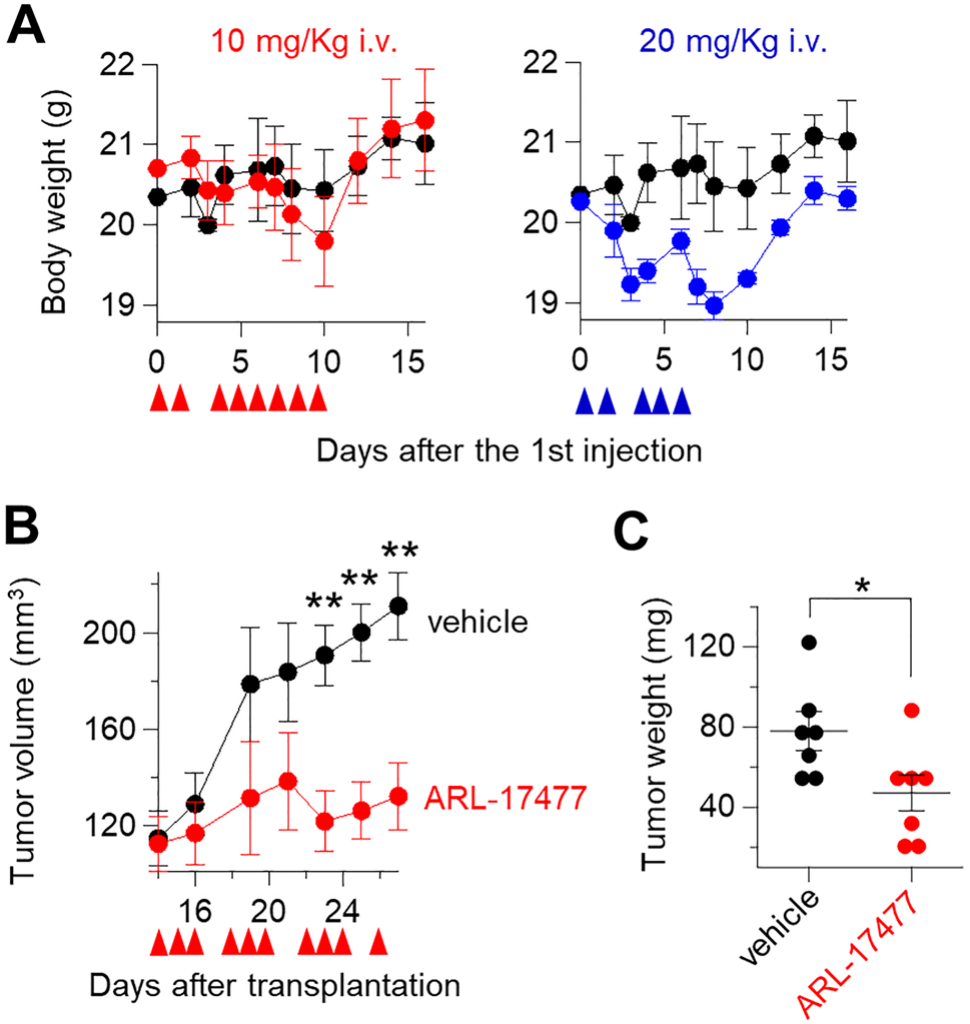
In vivo activity of ARL-17477. (**A**) Body weight changes of BALB/c mice (n = 3 mice per group) intravenously injected with vehicle (5% glucose) or ARL-17477 on days indicated by red arrows. (**B**) Tumor volume changes of MIA PaCa-2 xenograft mice (n = 7 mice per group) intravenously injected with vehicle or 10 mg/kg ARL-17477 on days indicated by red arrows. (**C**) Final tumor weight measured on day 27. Statistical comparisons were performed using unpaired Student’s *t* tests for two tailed p value. *p < 0.05, **p < 0.01.

In attempt to translate our findings to cancer therapeutics, we examined the anticancer activity of ARL-17477 in vivo. Previous preclinical studies suggested that ARL-17477 can be used without side effects at doses of 1–10 mg/kg via intravenous administration^2,4^. In addition, a previous pharmacokinetic study indicated that plasma concentration peaks at 0.16 µM at 1 h after the 1 mg/kg intravenous administration and decreases rapidly within the next 1 h^4^. Based on these preclinical data, we hypothesized that intravenous administration of 10 mg/kg ARL-17477 would reach a micromolar plasma concentration sufficient to suppress tumor growth (Table 1). Indeed, we confirmed that 10 mg/kg is the maximum tolerable dose for daily administration of ARL-17477; at doses > 20 mg/kg, mice lost > 10% of body weight during treatment (Fig. 6A). We transplanted MIA PaCa-2 cancer cells, a KRAS-mutant pancreatic cancer cell line most vulnerable to ARL-17477 (Table 1), subcutaneously into the tissue of nude mice. Treatment with 10 mg/kg ARL-17477 significantly delayed the tumor growth of MIA PaCa-2 cancer (Fig. 6B,C). Thus, ARL-17477 is an anticancer agent available in vivo.

## Discussion

Despite the widespread use of ARL-17477 as a selective NOS1 inhibitor in many preclinical studies, whether it demonstrated NOS1-indepenedent pharmacological activity was not previously known. The present study demonstrates, for the first time, that ARL-17477 can inhibit the autophagy-lysosomal system to prevent tumor growth in vitro and in vivo.

**Figure 7:**
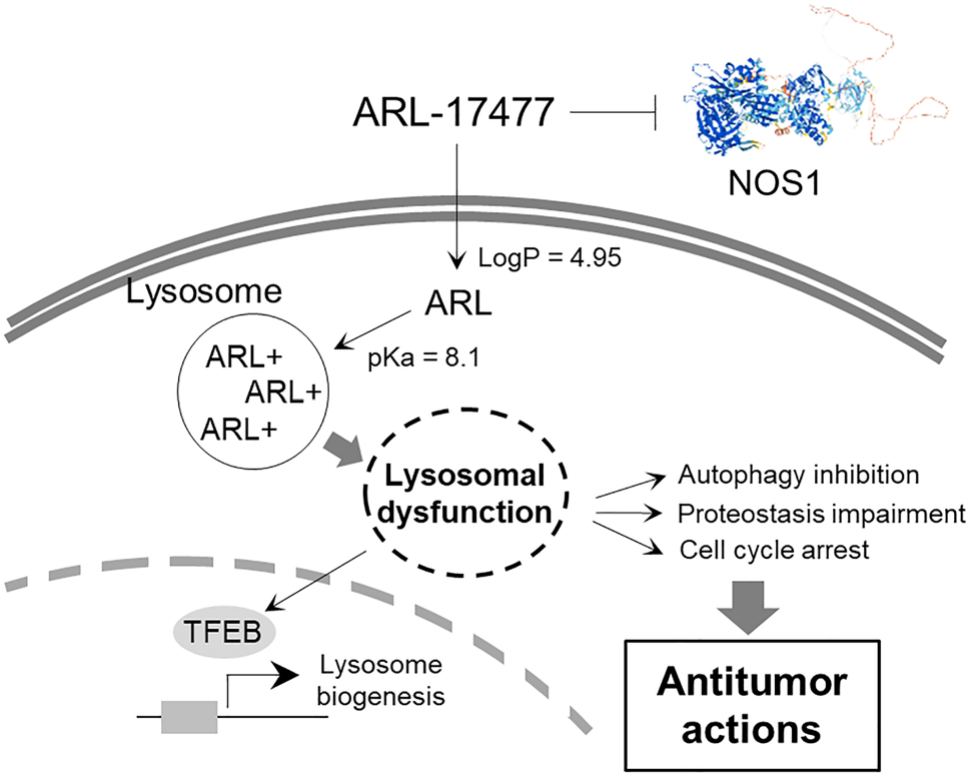
A NOS1-independent anticancer action of ARL-17477 involving inhibition of the autophagy-lysosomal system.

Figure 7 summarizes a plausible mechanism of the anticancer activity of ARL-17477. Initially, ARL-17477 diffuses through the cell membrane and distributes throughout the cytosol, due to its relatively high hydrophobicity (LogP: 4.95). However, once ARL-17477 reaches the acidic environment of the lysosome, its basic amine is protonated (pKa: 8.1), and its membrane permeability is significantly reduced. Consequently, ARL-17477 accumulates in the lysosome at a high concentration, possibly at a millimolar concentration similar to CQ^38^, to cause lysosomal dysfunction. We demonstrated the importance of lysosomal acidity to ARL-17477 activity using Bafilomycin A1, which increased lysosomal pH and thereby alleviated the effects of ARL-17477 on autophagy flux and lysosomal function (Fig. 4B,H).

We characterized ARL-17477-induced lysosomal dysfunction using lysosomal staining and the NanoBiT split luciferase assay (Fig. 4). Both assays consistently showed the occurrence of lysosomal dysfunction; however, lysosomal staining indicated that lysosomal dysfunction occurred even at 1-µM ARL-17477 (Fig. 4D), a concentration 100-fold lower than that indicated by the results of the NanoBiT assay (Fig. 4G). This large discrepancy partly arose from the size of pores formed in the lysosomal membrane, i.e. ARL-17477 at a few micromolar concentration might induce formation of small pores that allow the translocation of small molecules, such as Lysotracker, but not of large protein molecules, such as Pen-cRaf-v1^34^. Alternatively, ARL-17477 at a few micromolar concentration might increase lysosomal pH without inducing LMP, because Lysotracker and acridine orange are both pH-sensitive dyes that require acidic pH for lysosomal staining. Both scenarios suggest that ARL-17477 may perturb lysosomal function at a few micromolar concentration, either by decreasing pH or affecting membrane integrity.

Because lysosomes are essential for many cellular processes, their impairment induces a cascade of cellular events leading to cell death and cell cycle arrest^34^. In the present study, ARL-17477 was shown to impair at least two major cellular processes, autophagy and proteostasis (Figs. 3C and 4E). Specifically, ARL-17477 increased LC3B-II, p62, and GABARAP-II protein expression, indicating inhibition of autophagy flux at the level of autophagosome-lysosome fusion. We excluded the possibility of autophagy induction as a cause of this protein expression change, because cotreatment with ARL-17477 and Bafilomycin A1 alleviated the increase in autophagic protein expression (Fig. 4B).

Considering whether the inhibition of autophagic flux contributes to the anticancer mechanism of ARL-17477 is worthwhile. This research question has been studied in terms of the anticancer mechanism of CQ, and autophagy-deficient cell lines have been used to show that autophagy inhibition is not a direct cause of CQ-induced cell death^35^. Rather, parallel impairment of cellular processes with cytotoxic responses induced by LMP are considered responsible for the anticancer activity of CQ^39^. This parallel pathway includes not only impairment of autophagy but also lysosome-to-nucleus signaling impairment, ROS generation, and release of lysosomal enzymes. The same scenario could be applicable to the mechanism of ARL-17477-induced cell death.

Using microarrays and GSEA, we showed how cancer cells adapt to lysosomal dysfunction induced by ARL-17477 (Fig. 5). Notably, TFEB, a master regulator of autophagy-lysosome function, and lysosomal biogenesis were activated by ARL-17477. Activation of TFEB has been reported in several studies as a cellular adaptive response to lysosomal dysfunction^37,40–42^. Also, we identified the upregulation of genes related to lipid and cholesterol synthesis. Previous limited studies also reported stimulation of phospholipid and cholesterol synthesis by lysosomal inhibitors^43,44^, but the physiological significance of such stimulation remains unknown. A plausible scenario is that this may be an adaptive response of cells to compensate for decreased recycling of lipids, given that the lysosome is the site of the lipid salvage/recycling pathway^45^. Consistent with this scenario, TFEB is known to activate cholesterol and fatty acid synthesis^46^. Thus, upregulation of lipid and cholesterol synthesis may be a common cellular response to lysosomal inhibitors.

Taken together, our present findings suggest that lysosomes serve as the primary targets of ARL-17477 in terms of its anticancer activity (Fig. 7). This notion is supported by (1) similar IC_50_ values between cell viability and lysosomal function (Table 1 and Fig. 4D) and by (2) the basic amine of ARL-17477 that is essential for lysosomal accumulation (Fig. 4A). Meanwhile, we demonstrated that in vivo ARL-17477 inhibits the tumor growth of KRAS-mutant cancer. In combination, these results lead us to question whether lysosomes could serve as therapeutic targets in cancer treatment. Although targeting the lysosome is not a cancer-specific approach per se, we found that CSCs and KRAS-mutant cancer cells were more vulnerable to ARL-17477 than were other cancer cells (Table 1 and Fig. 1). KRAS-mutant cancer cells are known to demonstrate high levels of basal autophagy and thus might be more vulnerable to autophagy inhibition^47,48^. Also, CSCs are strongly dependent on autophagy both for survival and for their stem-like properties^49,50^, and several CSC-targeting drugs inhibit the autophagic-lysosomal pathway^51^. Thus, lysosomes could serve as a therapeutic target in the treatment of KRAS-mutant cancers and CSCs.

Finally, our findings suggest that ARL-17477 could demonstrate unique characteristics as a drug. Of note, there are many drugs acting as lysosomal inhibitors (more than 20), because any weak base capable of penetrating the cell membrane can accumulate in lysosomes and inhibit lysosomal function^28,52^. For example, a previous study demonstrated that 28 of 1024 approved drugs were possible inhibitors of the autophagy-lysosomal pathway^53^. Among the many possible lysosomal inhibitors, ARL-17477 is of interest because it inhibits both the lysosome and NOS1. Although not investigated in the current study, we anticipate that coinhibition of NOS1 and lysosomes will be beneficial to cancer treatment because (1) overexpression of NOS1 is observed in many tumors and considered responsible for in vivo tumor immune escape and metastasis^20,22^, and because (2) NOS1 inhibitor protects the brain from ischemic damage^1^, which can be induced by cancer-associated events and chemotherapies. This hypothesis should be investigated in future studies using immune-competent animal models and/or orthotropic xenograft models of brain tumors. Furthermore, strong penetrance of the blood–brain barrier with the establishment of safety and pharmacokinetic data would support the use of ARL-17477 as an anticancer therapeutic targeting brain tumors.

## Methods

### Reagents

The origins of cell lines and antibodies are listed in Table S2. All cell lines were authenticated by DNA short tandem repeat profiling by the suppliers or by Eurofins Genomics. All cell lines were used within 20 passages and confirmed to be mycoplasma negative using MycoAlert (Lonza # LT07-118). The KRAS mutation data were derived from the Cellosaurus website. ARL-17477 was purchased from Sigma-Aldrich (#SML0178) or FUJIFILM Wako (#517-93851).

### Generation of NOS1^−/−^ cell lines

Guide RNA (gRNA) against NOS1 gene was designed using the CHOPCHOP website (http://chopchop.cbu.uib.no/) and purchased from Integrated DNA Technologies (IDT). U251-MG cells were transfected with the gRNA and HiFi-Cas9 (IDT, #1081060) using Neon transfection system (Thermo Fisher Scientific). Single cells were collected on 96-well plates (1 cell/well) by a cell sorter and clonally expanded. Genomic DNA was examined for NOS1 mutations and several independent mutant clones were recovered.

### Cell viability assay

Cells were plated in Nunclon Delta-treated 96 well plates (Thermo Fisher Scientific #167425) with 10% fetal bovine serum (FBS)-containing Eagle’s Minimum Essential Medium (EMEM). The next day, attached cells were treated with a medium containing a compound for indicated times and cell viability was measured using Cell Counting Kit-8 (Wako #343-07623) with the manufacturer’s protocol.

### CSCs viability assay and sphere formation assay

Human patient-derived glioblastoma CSCs (TGS-01) were established and sphere formation assay was performed as described previously^54^. Osteosarcoma CSC (143B-sph) was established from 143B cell lines by sphere culture method; 143B spheres were cultured in Dulbecco’s Modified Eagle Medium/F12 serum-free medium supplemented with B27, 20 ng/ml of epidermal growth factor, and 20 ng/ml of basic fibroblast growth factor. For viability assay, 143B-sph were plated in 96-Well Nunclon Sphera-Treated plates. The next day, cells were treated with a medium containing a compound for 2 days and cell viability was measured using Cell ATP Assay reagent Ver.2 (Wako #387-09301). Prior to the ATP assay, spheres were rigorously disrupted by pipetting.

### Western blotting

U251-MG or MIA PaCa-2 cells were plated in 24-well plates with EMEM + 10% FBS and left overnight to adhere. Then, attached cells were treated with a medium containing a compound. After indicated times of incubation, cells were washed twice with cooled PBS, lysed by 1% SDS, and sonicated using an ultrasonic processor. The protein concentration of the cell lysate was measured using DC protein assay (Biorad #5000112JA). After equalizing the protein concentration in all samples, the lysate was analyzed by western blotting as previously described^31^.

### Lysosome and PROTEOSTAT dyes staining

MIA PaCa-2 cells were plated in 24-well glass-bottom plates with EMEM + 10% FBS and left overnight to adhere. Then, 50–80% confluent cells were treated with a medium containing a compound for 30 min, followed by treatment with 5 μg/mL acridine orange or 75 nM Lysotracker Red DND-99 for 30 min. The cells were washed twice with PBS and phenol-free DMEM was provided in the presence or absence of 5 μg/mL Hoechst 33532 for nucleus staining. Measurement was performed on BZ-X810 (Keyence). For cell cytometer analysis, Lysotracker-stained cells were trypsinized with 0.25% trypsin–EDTA for 5 min at 37 °C, and washed twice with phenol-free DMEM. Red fluorescence of the cells was measured using Tali Image Cytometer (Thermo Fisher Scientific). PROTEOSTAT staining (ENZ-51035-K100) was performed on MIA PaCa-2 cells 5 days after the treatment with ARL-17477 or CQ according to the manufacturer’s protocol.

### NanoBiT assay

MIA PaCa-2 cells were plated in 24-well plates with EMEM + 10% FBS, left overnight to adhere, and transfected with LgBiT-KRAS-G12V using Lipofectamine 3000 Transfection Reagent (Thermo Fisher Scientific #L3000001) as previously described^31^. After overnight incubation, the cells were detached by trypsinization, reseeded into 96-well CELLSTAR plate (Greiner #655083), and left overnight to adhere. Then, 30–50% confluent cells were treated with a medium containing a compound for 1 h, followed by treatment of 5 µM Pen-cRaf-v1-SmBiT for 1 h. The cells were added with Nano-Glo Live Cell Reagent (Promega #N2011) for 5 min, and luminescence was measured by SpectraMax® iD5 (Molecular Devices).

### Microarray and GSEA

Panc-1 cells were treated with a medium containing 25 µM ARL-17477 or vehicle only for 48 h. Extraction of RNA and expression profiling were performed at Gifu University Division of Genomics Research using Agilent SurePrint G3 Human GE 8 × 60 K Ver. 3.0 Microarray. For GSEA, gene expression data were queried against MSigDB v7.5.1 databases for the collections of KEGG pathway gene sets (C2_CP:KEGG)^55,56^ and regulatory target gene sets (C3_TFT) with 1000 permutations (gene set) and default parameters on GSEA 4.2.2.

### In vivo test

All mouse experiments were approved by Committee for Animal Research and Welfare of Gifu University (approval no. 2021-142) and performed in accordance with institutional welfare guidelines. The study is reported in accordance with ARRIVE guidelines (https://arriveguidelines.org). A cell suspension containing 10^7^ MIA PaCa-2 cancer cells in 100 µL PBS was subcutaneously inoculated into the right flank of 6 to 8-week-old female BALB/c-nu/nu mice purchased from Japan SLC. 14 days after the inoculation, mice were randomized (n = 7 mice per group) and treated with vehicle control (5% glucose) or ARL-17477 by an intravenous bolus via a tail vein at the indicated dose. Tumor volumes were calculated by the formula, 0.5 × width^2^ × length. For toxicity study, BALB/c mice without tumors were treated with ARL-17477 as described above, and behavior abnormalities and changes in body weight were evaluated.

## Supplementary Information

Supplementary Figures.

Supplementary Table 1.

Supplementary Table 2.

## Data Availability

The datasets used and/or analyzed during the current study available from the corresponding author on reasonable request.
