# Supplementary material for: ARL-17477 is a dual inhibitor of NOS1 and the autophagic-lysosomal system that prevents tumor growth in vitro and in vivo
Source: Sci Rep. 2023 Jul 4;13:10757. doi: 10.1038/s41598-023-37797-4 (PMC10319805; doi:10.1038/s41598-023-37797-4)
Supplement: Supplementary file 1 — Supplementary Figures. [file 41598_2023_37797_MOESM1_ESM.pdf]

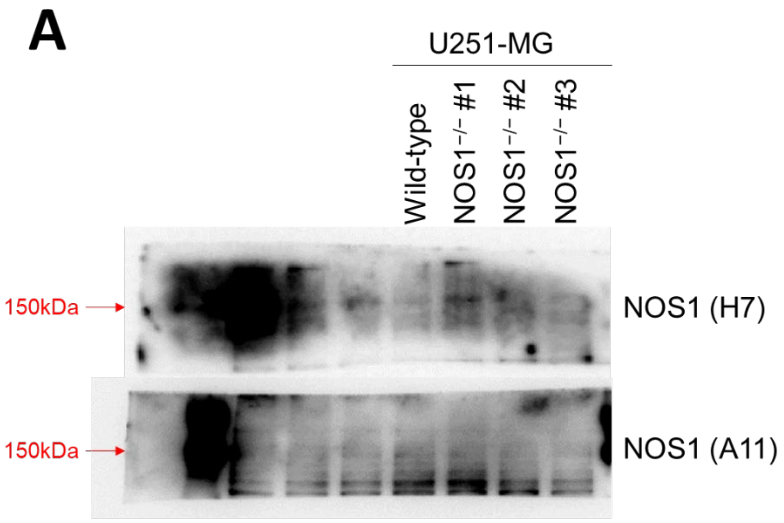

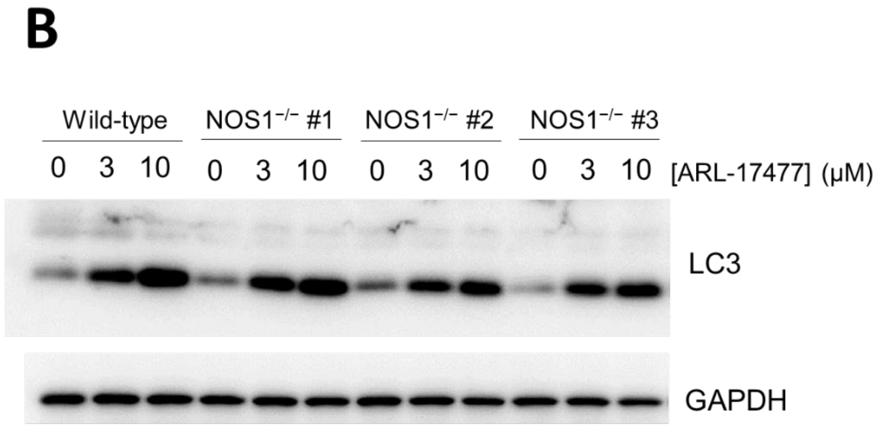

Figure S1. Immunoblots of U251-MG wild-type and knockout cells following treatment with ARL-17477. Original blots are presented in Supplementary Figure S5.

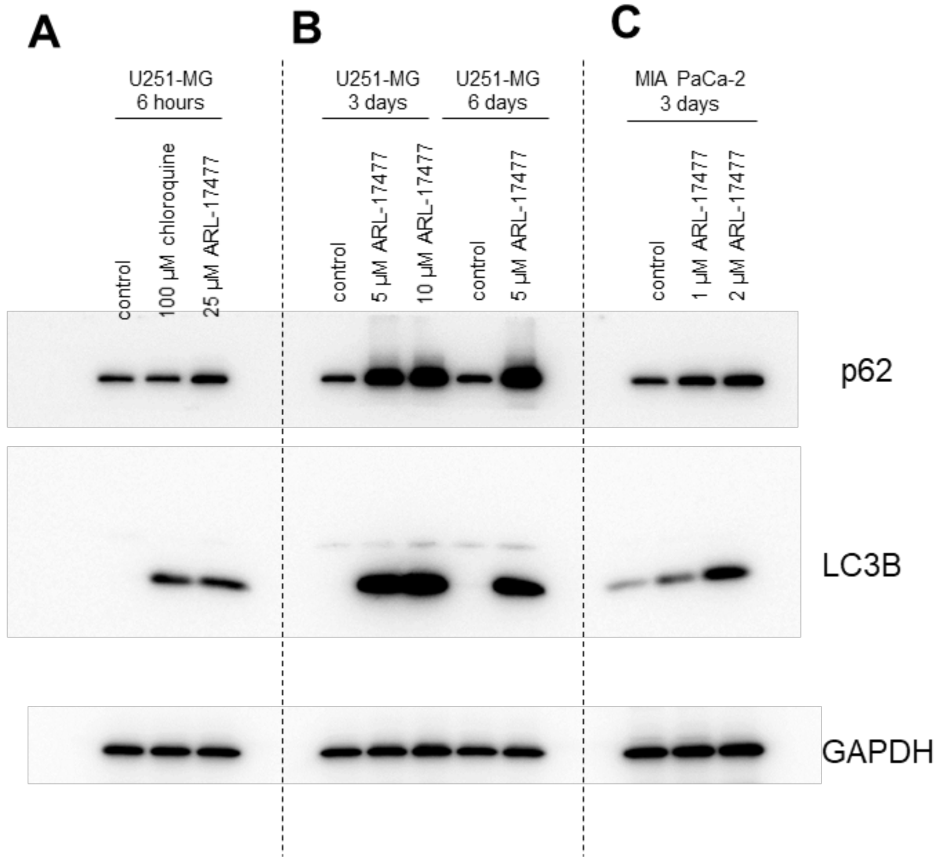

Figure S2. Immunoblots of cancer cells following treatment with ARL-17477 or chloroquine. Original blots are presented in Supplementary Figure S6.

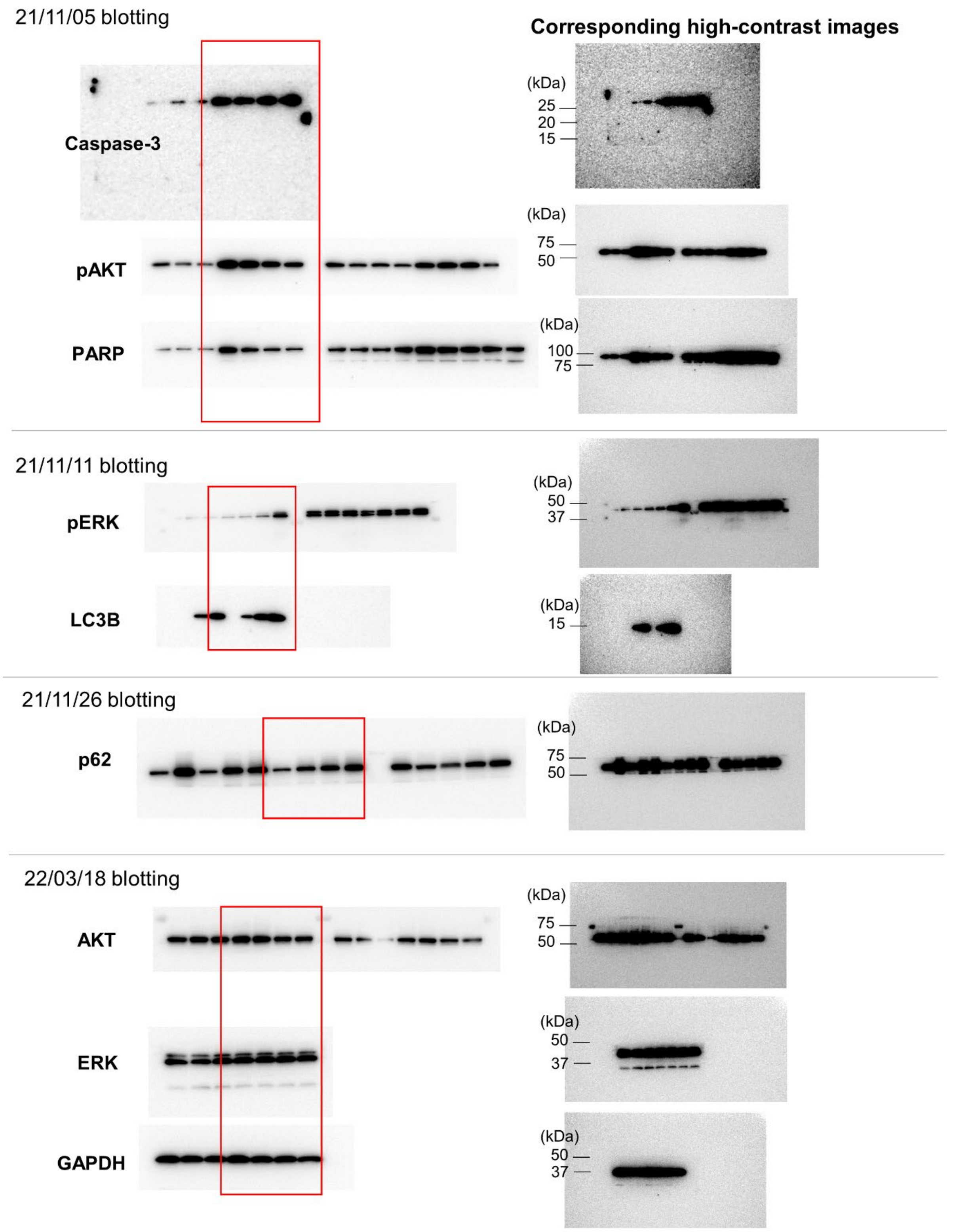

Figure S3. Original immunoblot images for Figures 3A and 3C. Note. all blot membranes were cut into stripes prior to hybridization with antibodies. Membrane edges are not clear in some cases, due to high signal-to-noise ratio of luminescence intensity.

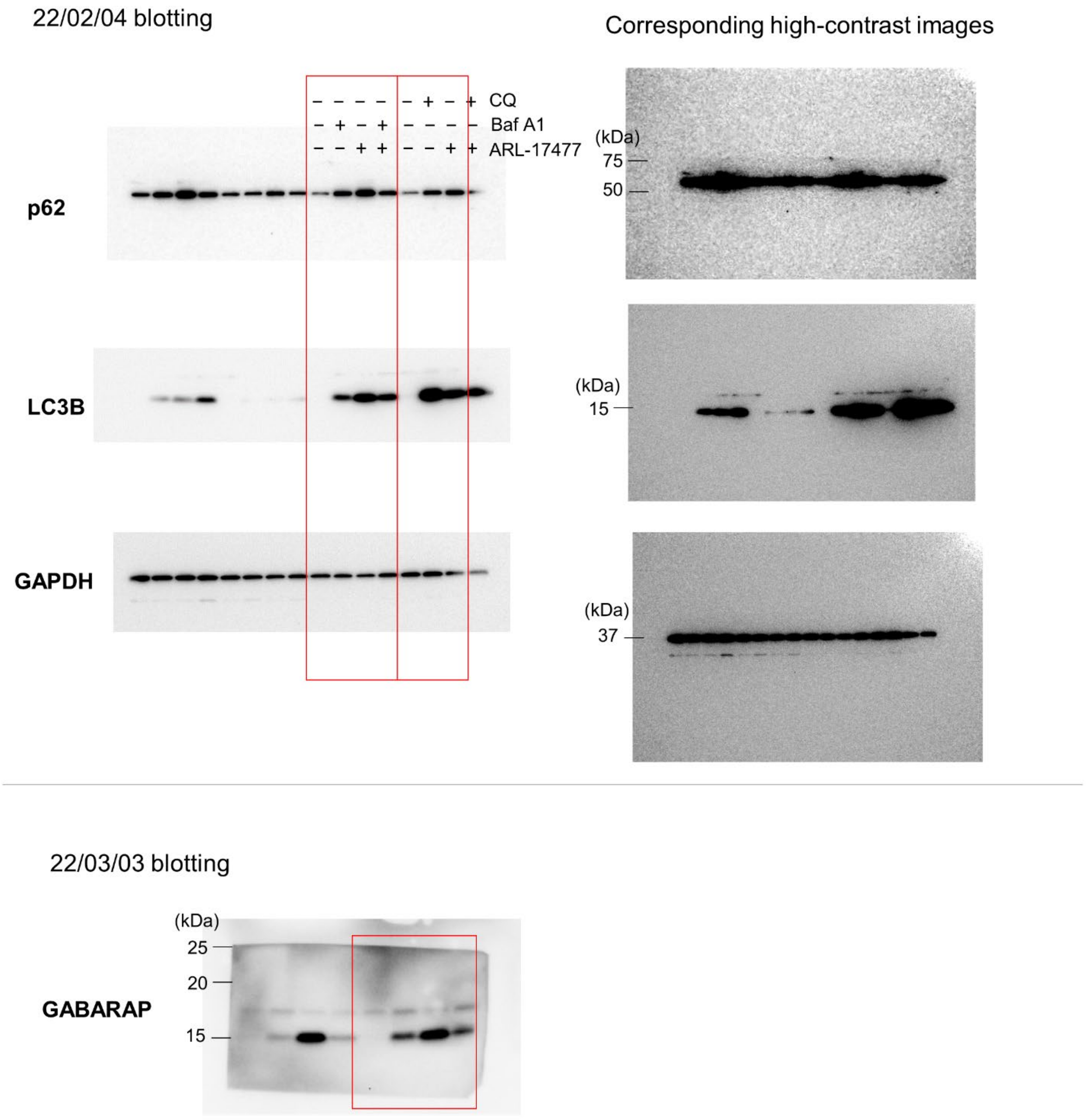

Figure S4. Original immunoblot images for Figures 3D and 4B. Note. See Legend of Figure S3 for membrane processing.

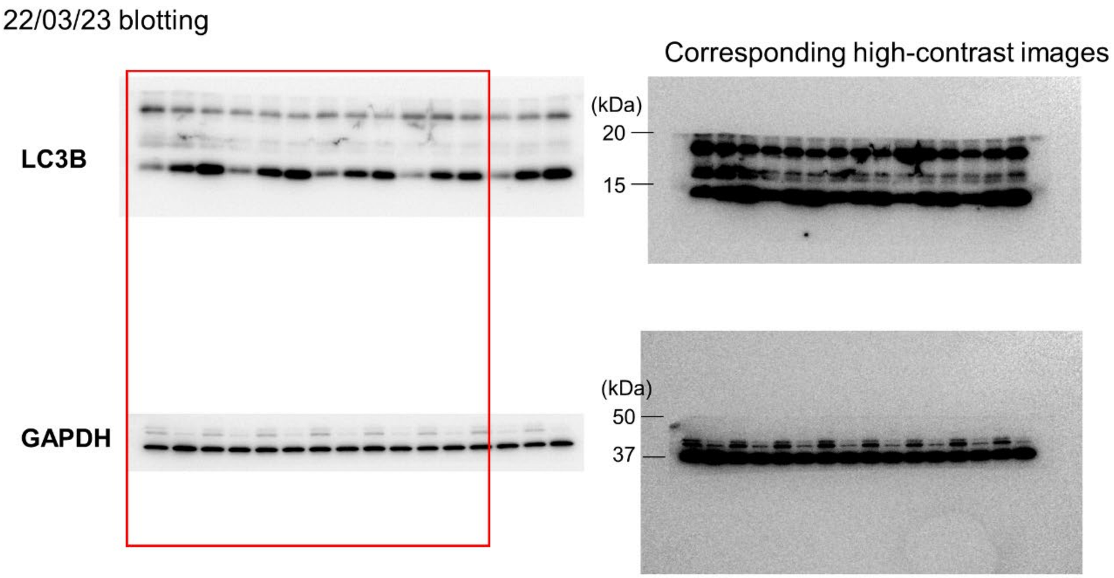

Figure S5. Original immunoblot images for Figure S1. Note. See Legend of Figure S3 for membrane processing.

22/11/17 blotting

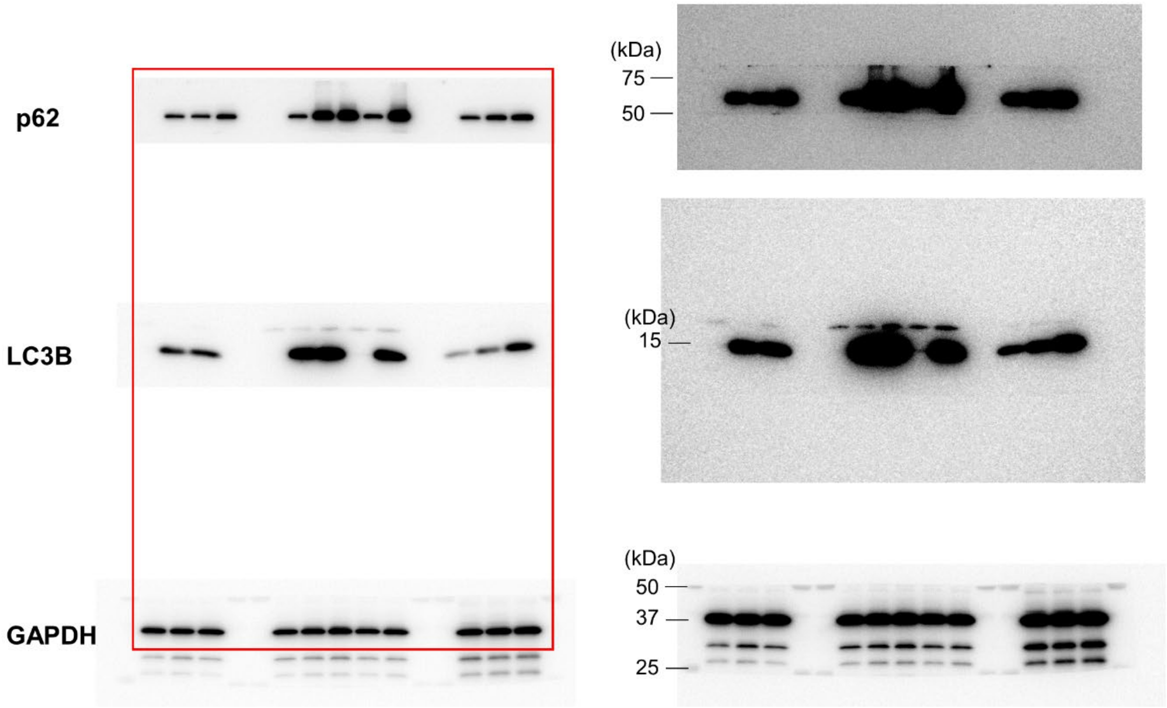

Figure S6. Original immunoblot images for Figure S2. Note. See Legend of Figure S3 for membrane processing.
